# Role of microRNAs in myogenesis and their effects on meat quality in pig — A review

**DOI:** 10.5713/ajas.20.0324

**Published:** 2020-08-19

**Authors:** Ambreen Iqbal, Jiang Ping, Shaokat Ali, Gao Zhen, Liu Juan, Jin Zi Kang, Pan Ziyi, Lu Huixian, Zhao Zhihui

**Affiliations:** 1 Department of Animal Breeding and Genetics, College of Coastal Agricultural Sciences, Guangdong Ocean University, Zhanjiang, Guangdong 524088, China

**Keywords:** microRNA, Myogenesis, Meat Quality, Pig

## Abstract

The demand for food is increasing day by day because of the increasing global population. Therefore, meat, the easiest and largely available source of protein, needs to be produced in large amounts with good quality. The pork industry is a significant shareholder in fulfilling the global meat demands. Notably, myogenesis-development of muscles during embryogenesis- is a complex mechanism which culminates in meat production. But the molecular mechanisms which govern the myogenesis are less known. The involvement of miRNAs in myogenesis and meat quality, which depends on factors such as myofiber composition and intramuscular fat contents which determine the meat color, flavor, juiciness, and water holding capacity, are being extrapolated to increase both the quantity and quality of pork. Various kinds of microRNAs (miRNAs), miR-1, miR-21, miR22, miR-27, miR-34, miR-127, miR-133, miR-143, miR-155, miR-199, miR-206, miR-208, miR-378, and miR-432 play important roles in pig skeletal muscle development. Further, the quality of meat also depends upon myofiber which is developed through the expression of different kinds of miRNAs at different stages. This review will focus on the mechanism of myogenesis, the role of miRNAs in myogenesis, and meat quality with a focus on the pig.

## INTRODUCTION

The world’s population has increased very rapidly in recent years. According to an estimate, the world’s population in 1915 was approximately 1.8 billion. A recent survey by the United Nations (UN) estimated the world’s population at 7.7 billion in 2019 will reach 9.7 billion by 2050 [1]. Over the years, the dietary habit of most people has changed owing to increase in population and changes in economy, especially in developing countries. This accounts for a high increase in demand for animal meat and protein. To meet the ever-increasing food demand requires more research for improving the existing food production system. The development of skeletal muscle is a complicated process comprised of embryonic myogenesis to postnatal muscle growth. The postpartum muscle growth in swine is dependent on the total number of myofibers which are generated in two waves before birth. Pig myogenesis occurs in two phases, the first phase includes the formation of primary myofibers from 35 to 55 days post coitus (dpc), followed by the formation of secondary myofibers around each primary myofiber between 50 and 90 dpc [2]. Postpartum muscle growth is generally considered from birth to 60 days. Postnatal muscle growth mostly includes the conversion from slow-oxidative to fast glycolytic fiber types [3].

Meat quality is an important commercial trait in domestic animals, which is mainly estimated by intermuscular fat (IMF), pH value, tenderness, flavor substance, and water holding capacity. Further, meat quality is significantly affected by myofibers composition [4]. Quality of pork is mainly based on different kinds of myofibers and different contents of IMF present in different swine breeds. The pork industry mainly focuses on enhancing the muscle mass and development rate [5]. This goal achieved in various breeds of swine through genetic selection and cross-breeding [6]. Through these tactics, the Landrace swine breed has been developed, which has high body weight, rapid growth ability, and high lean meat percentage [7]. Despite Landrace, some local breeds have less weight and a slow growth rate, but these local breeds have high IMF content that affects the flavor of meat [8].

MicroRNAs are short (approximately 22 to 25 nucleotide), non-coding, single-stranded RNA molecules that are responsible for controlling gene expression through translational degradation, repression, and deadenylation of target mRNA. Studies present strong evidence that miRNAs play important roles in porcine skeletal muscle development [9–18]. The most important miRNA in myogenesis is miR-133a [19], which plays regulatory roles in muscle tissues [20–22]. Both miR-1 and miR-133 have multiple functions in myogenesis primarily, they are responsible for skeletal muscle development, and secondarily, they also act as myocyte enhancers [20]. Due to the advancement in sequencing technologies the numbers of miRNA discovered increased day by day [23]. Different type of miRNAs found to play regulatory roles in pig skeletal muscle development includes miR-1, miR-21, miR22, miR-27, miR-34, miR-127, miR-133, miR-143, miR-155, miR-199, miR-206, miR-208, miR-378, and miR-432 [12,14,15,17,20,23–26]. Here, we will discuss the regulatory roles of different miRNAs in myogenesis and meat quality regulation in pigs.

## PRINCIPLES OF MYOGENESIS AND POSTNATAL MUSCLE GROWTH

Prenatal swine myogenesis depends on the number of factors including nutrient accessibility, hormones, and growth factors that influence the changes in metabolism at the transcriptional and translational level of regulatory genes. These factors, linked with the myogenic regulatory factors, play a significant role in controlling muscle-specific gene expression [27]. Skeletal muscle development in pigs occurs in two phases, the first phase occurs from 35 to 55 dpc in which the primary myofibers are formed and the formation of secondary myofibers around each primary myofiber occurs between 50 and 90 dpc [2,28]. In prenatal development of muscles, the content of fibers depends on the propagation and apoptosis of stem cell differentiation and the conjoining of myoblasts accompanied by the ultimate maturation of myofibers. Myostellite cells, with multipotency, can differentiate to muscle cells, but are not able to form the myofibers. During postpartum growth, these cells act as the source of new myonuclei which play a major role in fiber size and regeneration process. Despite this contribution, myonuclei remain mitotically dormant. The number of fibers is determined during the prenatal development stage in most of the mammalian species and remain fixed throughout life. Albeit, under the influence of the genetic and physiological factors, the size of the fibers increases without an increase in the number of fibers [29]. During post-partum muscular development, if the fiber numbers are more, then the individual fiber grows slowly and vice versa [29]. In an adult, the muscle’s cross-sectional area directly relates to the number and size of fibers. One such example is of massive loin area which is composed of, in most cases, the low fiber size and high fiber number and vice-versa. However, the best loin area can be achieved through an optimal combination of fiber size and fiber number.

## MOLECULAR MECHANISMS OF MYOGENESIS AND INDICATORS OF MEAT QUALITY

In the late 1980s, the revolutionary discovery of the myoblast differentiation (*MyoD*) family of basic-helix-loop-helix transcription factors responsible for regulating the molecular mechanism of muscle development has opened a new arena of discussion. The four master members of the *MyoD* family, myogenic regulatory factors (*MRFs*), *MRF4*, myogenin (*MyoG*), and myogenic factor 5, play key roles in myogenesis. Regarding their origin, different parts of the muscles have different origins. For example, trunk and limb muscles are engendered from somite-derived dermomyotome. While extraocular, neck, and head muscles originate from cranial mesoderm [30]. During myogenesis, microsatellite cells pass through several processes regulated by vital transcriptional factors giving rise to myoblast cells which, ultimately, differentiates to form myofibers [31]. From the surrounding environment, extracellular signaling molecules released are bone morphogenetic protein (*BMPs*), wingless and Int (*Wnts*), and sonic hedgehog (*Shh*) responsible for sclerotome differentiation and determination [32]. Transcription factors produced from these signaling proteins affect stem cells responsible for the determination of the myogenic fate. These signaling molecules are released from different parts, but all have effects on sclerotome development. *BMP* and the *Shh* are released from the notochord through transcription factor paired box protein (*Pax1*), which plays a significant role in sclerotome development. *Pax1* is responsible for the changes in sclerotome progenitor cell’s epithelium, which ultimately differentiate into chondrocytes. *Wnt1* and *Wnt3a* determine the myogenic fate of epaxial dermomyotome, both of which are secreted along with *Shh*. *Shh* is secreted from the floor plate of the neural tube and the notochord, while *Wnt1* and *Wnt3a* are released from the dorsal neural tube. *Wnt7a* is responsible for specifying the myogenic fate of hypaxial dermomyotome which is released from the dorsal ectoderm [33].

The quality of meat mainly depends on the type of myofibers. A myofiber is divided into four types due to the presence of the phenomenon of polymorphism in the myosin heavy chain. The type is I with myosin heavy chain (*MyHC*) I, type IIa with *MyHC IIa*, type IIx with *MyHC IIx*, and type IIb with *MyHC IIb* [34]. The type I and IIa fibers are included in slow and fast oxidative muscle types, while type IIx and IIb fibers are considered intermediate and fast glycolytic myofibers. If we compare the properties of both fiber types, then we can conclude that type I and type II have higher myoglobin content, more mitochondria, low glycolytic capacity, and less myosin ATPase activity. The type IIb fibers have more glycogen, longer fiber size, and they show quick contractions. The pork flavor, tenderness, water holding capacity, and IMF are determined by type I and II type fibers, and these fibers also reduce the risk of soft, exudative, and pale pork efficiently [35,36].

## MicroRNAs AND THEIR ROLE IN MYOGENESIS

### Overview of miRNAs

MicroRNAs are short (approximately 22 to 25 nucleotides), non-coding, single-stranded RNA molecules that are responsible for controlling gene expression through translational degradation, repression, and deadenylation of target mRNA. The degradation of the target mRNA to terminate protein translation is achieved through the binding of miRNA with RNA-induced silencing complex (RISC) at the 3′untranslated region (3′ UTR) [37]. These small non-coding RNAs play significant roles in different biological processes, with inevitable roles during adipogenesis [38] and myogenesis [39]. RNA polymerase II plays a major role in the transcription of miRNA into pri-miRNAs [40]. Then pri-miRNA is wrapped into a hairpin structure and further processed by a nuclear endonuclease RNAIII, Drosha, which on an average adds 70 to 90 nucleotide precursors and forms the pre-miRNAs [41]. After that, pre-miRNA transfers to the cytoplasm at the place where Dicer is present which belongs to the RNase III endonuclease, this is also responsible for the conversion of the pre-miRNA into mature miRNA [42]. Before the degradation of mature miRNA, miRNA combines with Argonaute protein and forms the RISCs. After the RISCs formation, mature miRNAs are degraded [43]. The activity of the RISCs is the same as that of exonuclease and endonuclease. RISC plays a crucial role in both translation and stability of mRNA [44]. Studies suggest that the earliest miRNA discovered in *Caenorhabditis elegans* gene having 22nt [45] was named as let-7 comprised of 22nt [46]. With time, thousands of miRNAs were recognized in different species. According to the miRbase, 21.0 total 28,645 miRNA were estimated (http://www.mirbase.org/). In different species, different kinds of miRNA prophesied and confirmed. The total number of miRNA present in humans is 2,588 [47], in mice is 1,915 [48], in bovine is 793 [49] and in sheep, the number of respective miRNAs is just 153 [50,51].

### Role of miRNAs in myogenesis

The miRNAs play significant roles in the physiological processes including proliferation, apoptosis, and differentiation and all of these steps have a direct influence on embryogenesis, development timing, organogenesis, growth control, and cell linage [52]. miRNAs play significant roles in myogenesis which involves different steps including muscle cell proliferation and differentiation, determination of myofibers, and skeletal muscle atrophy or hypertrophy. During normal muscle growth, miRNA, and the gene coding for proteins interlinking with each other show the Spatio-temporal expression patterns [53]. Previous studies reported that miRNAs play major roles in porcine myogenesis [23,54–56].

Skeletal muscles play significant roles in energy metabolism, exercise [57], and are the main component of meat. Based on weight, skeletal muscles occupy a total of 40% of body mass. The composition of skeletal tissue is heterogeneous and comprised of different kinds of fibers. These fibers are generally divided into three categories, glycolytic (white fibers), oxidative (red fibers), and oxidative-glycolytic (intermediate fibers). The division of fibers is based on various kinds of *MyHC*. White fibers have *MYH1*, red fibers have *MYH7*, and intermediate fibers have *MYH2* [35,58]. In a comparative scenario of red and white fibers, red fibers have more myoglobin, lipids, capillaries, and mitochondria as compared to white fibers. While the white fibers have a higher level of lactate dehydrogenase A, which is the master glycolytic enzyme in skeletal muscles [56].

With the advancement in knowledge, it is suggested that glycolytic and oxidative skeletal muscles have the same miRNA expression during different stages of development due to their metabolic requirements [59,60]. Recently, Chen et al. analyzed the expression of miRNAs in the longissimus dorsi of Chinese native Rongchang pigs at weaning and slaughter time. They found 19 novel and 186 previously known miRNAs related to the skeletal muscle development in the Rongchang pig. Porcine miR-27, miR-299, and miR432-5p showed downregulated expression patterns in adult pigs. While in weaning pigs, miR-7134-3p and 664-5p were significantly upregulated. These miRNAs which playing significant roles in skeletal muscle development could be used as biomarkers after further validation. Further, the authors also reported that miR-217 could act as an inhibitor of porcine satellite cells (PSCs) proliferation and differentiation through downregulating the expression of *cyclin B*, *cyclin D*, as well as proliferating cell nuclear antigen (*PCNA*) and *MyoG* and *MyHC* respectively [61]. Remarkably, miR-208b and miR-499 are responsible for the transcriptional repression of the slow-twitch protein gene *SRY*-box transcription factor 6 (*Sox6*). So, both of these miRNAs are directly linked with the oxidative red fibers [62]. However, porcine miR-208b also regulates the expression of *Sox6* and *MYH7* which effects the myofibers characteristics and pork quality [63]. For the differentiation event to take place, the arrest of the cell cycle and a decrease in DNA synthesis is critical [25]. In muscle cell differentiation, miR-206 plays its role in multiple ways, one being the inhibition of DNA polymerase alpha 1 (Pola1) which is the largest DNA polymerase subunit and controller of DNA synthesis. Inhibition of Pola1 leads to the cell cycle dormancy and starting of the differentiation of myoblasts in C2C12 cells [25]. Another mechanism through which miR-206 regulates the differentiation of skeletal muscle cells is the inhibition of *Pax3* and *Pax7* at particular timespan in development for promoting the terminal differentiation of myoblasts [64]. Both *Pax3* and *Pax7* are involved in cell survival and maintenance of myoblast and satellite cell proliferation [65,66]. Thus, the timely inhibition of these factors is necessary to start the differentiation of myoblasts which is achieved by the higher expression of miR-206 during embryonic development [64]. Further, the binding of myogenic factors, *MyoD* and *MyoG*, to the upstream regions of miR-206 and miR-133b, was evidenced through chromatin immunoprecipitation [67]. In addition to the above-mentioned mechanisms, miR-206 involves the enhancement of muscle cell differentiation by stopping the activity of the connexin43 (*Cx43*) gene responsible for muscle cell fusion [68]. Another study reported the combined effect of miR-1/206 in the regulation of porcine skeletal muscle development. The secreted frizzled-related protein 1 (*SFRP1*), inhibitor of *Wnt* signalling pathway, was found to be regulated by the miR-1/206 in porcine iliac endothelial cells [69].

Another important myomiR to play a similar kind of role like miR-206 is miR-1. Both miR-1 and miR-206 have the same seed sequence and target genes (*Cx43*, *Pax3*, and *Pax7*). But the miR-1 has more target genes and thus, plays more regulatory roles in skeletal muscle differentiation as compared to miR-206. miR-1 binds to the 3′UTR of class II histone deacetylase, histone deacetylase 4 (*HDAC4*) mRNA which inhibits the myocyte enhancer factor-2 (*MEF2*) protein expression required for enhancement of muscle development [20,70]. Additionally, miR-1 also inhibits the expression of ying-yang 1 (*YY1*), which plays a negative role in muscle gene transcription [71]. More advanced studies reported that miR-1 and miR-206 both are closely related miRNAs to play key roles in myogenesis in mouse myoblast cell line, C2C12. Several genes including SWI/SNF-related matrix-associated actin-dependent regulator of chromatin subfamily B member 1, 2, mitogen-activated protein kinase kinase kinase kinase 3, homeobox protein MOX-2, and frizzled-7 have a direct effect on miR-1 and miR-206 which triggers skeletal muscle development [72]. Further, miR-1 was also found to target the calponin 3 (*CNN3*) gene which is involved in skeletal muscle development in pigs [73]. Hong et al [74] reported two SNPs at the miR-1 locus in Berkshire, Landrace, and Yorkshire pigs. These SNPs were related to the type I and type IIa myofibers area and composition and caused the alteration in levels of miR-1 which is associated with regulating myofibers type determination [74].

Another myomiR to play significant roles in skeletal muscle development is miR-133. This miRNA occurs in two forms, miR-133a and miR-133b. Transcription factors like *MyoD*, *MyoG*, serum response factor, and *MEF2* act as the regulators of miR-1 and miR-133a [20]. miR-133 also shows the muscle-specific expression-pattern and regulates the expression of mastermind-like protein 1, insulin-like growth factor 1 receptor, and neural polypyrimidine tract binding protein for the differentiation of skeletal muscles. Metabolic properties associated with fiber type impact the conversion of muscles to meat and eventually to meat quality. Other miRNAs involved in porcine skeletal muscle development include miRNA-21, a novel myogenic miRNA that regulates skeletal muscle development through controlling the Pphosphatidylinositol-3-kinase/protein kinase B/mammalian target of rapamycin (*PI3K/Akt/mTOR*) signaling by targeting the transforming growth factor-β I (*TGFβI*) gene [18]. In PSCs in skeletal muscle miR-22 inhibits the proliferation through targeting the *cyclin D1*, *cyclin B1*, and *p21* but promotes the differentiation through regulating the expression of *MyoG* and *MyHC* [61]. Further, miR-27a inhibits the expression of *MyoG* and it also regulates the protein kinase B/forkhead box protein O1 (*Akt/FoxO1*) signal pathway during porcine myogenesis [75] while miR27b acts to promote PSCs myogenesis by targeting *MyoD* family inhibitor (*MDFI*) [76]. Besides, mi-34c, inhibits PSCs proliferation and promotes PSCs differentiation. miR-34c forms a regulatory loop with *Notch1* for repressing muscle development through inhibiting the proliferation of PSCs [77], ssc-miR-143-3p regulates the expression *MYH7* in skeletal muscle PSCs through the *HDAC4-MEF2* pathway for regulating myofibers differentiation in skeletal muscles [78], miR-155 acts through the regulation of olfactomedin-like protein 3 (*OLFML3*) for playing its major role in swine prenatal myogenesis [79], miR-199b regulates the porcine skeletal muscle satellite cell’s proliferation through a feedback loop with the *JAG1-Notch1* signal pathway [80], miR-378 acts through regulation of mitogen-activated protein kinase 1 (*MAPK1*), and bone morphogenetic protein 2 (*BMP2*) is responsible for porcine myogenesis [12]. Ren et al [81] analyzed the expression of miR-432 in skeletal muscles of embryonic and adult Landrace pigs. The expression of miR-432 was found to be higher in embryonic samples as compared to the samples from adult stages. Myozenin1 (*MYOZ1*) protein involved in the muscular sarcomere microstructure was targeted by miR-432, thus, helping in the myogenesis along with other myomiRs [81]. The summary of these miRNAs and their roles in porcine myogenesis along with their target genes is given in Table 1.

## ROLE OF miRNAs IN PORK QUALITY

### miRNAs and the development of meat fiber

Meat quality depends upon the meat composition comprised of the myofibers (lean to fat ratio) and other palatability factors such as its visual look, smell, juiciness, tenderness, and flavor. The most significant factor that affects meat quality is the type of fibers [82]. The myofibers are categorized into four groups based on MyHC polymorphisms. Myofibers comprised of type I having *MyHC I*, type IIa having *MyHC II*, type IIx having *MyHC IIx*, and type IIb having *MyHC IIb* [34]. Type I and type IIa fibers are fast oxidative or slow myofibers while type IIx and type IIb are fast glycolytic and intermediate myofibers. When we compare the property of type IIb with type I fibers it is noted that type I has more myoglobin and mitochondria content, low glycolytic capacity, myosin ATPase activity level. In contrast to that, type IIb fibers are enriched with more glycogen, and longer fiber size, so, they show a more rapid contraction speed. The fiber type I and type II are responsible for tenderness, flavor, water holding capacity and IMF of meat and also play a significant role in decreasing the production of exudative, pale and soft pork [35,83].

Transcription factors play a significant role in the determination of myofiber types. *Sox6* is an important transcriptional factor belonging to *Sox* (Sry-related high mobility group [HMG] box) family, which is extensively present in skeletal and cardiac muscles [84]. *Sox6* was first isolated from an adult mouse testis [85]. It plays significant roles in embryonic myogenesis and also responsible for the maintenance of mature myofibers [84,86]. The absence of *Sox6* causes a reduction in the differentiation of myofibers which makes it obvious that *Sox6* plays a crucial role in the formation of myogenic fibers [86].

The other members of the myomiRs include miR-208a, miR-208b, and miR-499-5p. These three myomiRs are highly homogenous and all exhibit myofibers identification and are responsible for the switching of myosin [87]. They play roles in the determination of myofibers and also hold the ability to regulate myosin switching. Fast skeletal muscle genes, Troponin T3 (*Tnnt3*), and Troponin i2 (*Tnni2*) were expressed in the heart upon the deletion of miR-208a [88]. Further, the deletion of miR-208b and miR-499-5p prompted the loss of type I myofiber [89]. In PSCs, the overexpression of miR-499-5p resulted in increased expression of *MyHCI* and *MyHC IIa* mRNA. This shows the importance of miR-499-5p in myofiber specification in pigs [90]. The advanced research in the field of molecular mechanisms regulating the development and transition of myofibers has explored the roles of miRNAs in these mechanisms. One such example includes the role of miR-499-5p in soleus and extensor digiorum longus myofibers determination of swine through regulation of *Sox6*. The miR-499-5p through downregulation of *Sox6* causes oxidative myofibers formation. This mechanism introduces a new theme in the betterment in pig meat quality, flavor, tenderness, and IMF.

Type IV collagen encodes from the collagen, type III, alpha 1 (*COL3A1*) gene [91]. Collagen IV is a master component of various tissues [92] and plays a significant role in smooth muscle integrity and tension conservation [93]. Intramuscular (IM) collagen plays a major role in meat quality including water holding capacity and tenderness [94]. The newly stated conserved miRNA-29 family acts as a master regulator in extracellular matrix homeostasis [95]. The miRNA-29 family includes miR-29a, miR-29b, miR-29c, and miR-29d. The miR-29 family has major roles in regulating extracellular matrix genes including collagen, so, it is responsible for the fibrosis of many organs [96]. In muscle, *COL3A1* has a great influence on intramuscular collagen content and characteristics [97]. In Laiwu pigs, higher expression of *COL3A1* produces more type IV collagen due to which its meat has a high water-holding capacity and good tenderness capacity [97].

## INTERLINKING OF miRNAs AND IMF IN THE CONTEXT OF PORK QUALITY

China is the biggest producer and consumer of pork. Pork consumers tend to consume pork with good IMF which is also a key indicator of pork quality [98,99]. The contents of IMF are used as a trait for quantifying the percentage of fat content in myofibers and it acts as an economic factor in the pork industry. It impacts the overall carcass quality not only through marbling appearance but also through affecting the tenderness and juiciness of meat [100]. Thus, the researchers are showing great interest to produce pork with high IMF contents. Thus far, the molecular mechanisms underlying the deposition of IMF are not clear. In recent years, some progress has been made in exploring the role of miRNAs in intramuscular preadipocyte development [101]. IMF deposition is a complex process that could be diverse among different species, breeds, age, and depends upon the nutrition [102–104]. The IMF content may vary from 2% to 10% in different breeds of swine [105]. But the understanding of the genetic and molecular players in IMF deposition could help in the selection and breeding of high IMF content animals. Therefore, in recent years, researchers have scratched the surface of this field for understanding the molecular mechanisms governing IMF deposition, but much more work is needed to establish the paradigm-shifting impact in improving the quality of pork production.

The expression of miRNAs was analyzed by Liu et al [99] in the longissimus dorsi muscle of Yorkshire (YY, lean-type) and Chinese Wannanhua (WH, fatty) pigs. They found 598 miRNAs from which 42 were differentially expressed between YY and WH pigs. Two miRNAs, miR-196a/b (miR-196a, miR-196b-5p), were highly expressed in WH pigs giving the clue about their influence in porcine adipogenesis an adipocytokine signaling pathways [99]. The differential expression of miRNAs between IM and subcutaneous (SC) adipocytes from Jiaxing black pigs, one of the Taihu pig breeds well known for its high IMF and excellent pork flavor [106], showed 155 miRNAs with significant differential expression (SDE). Target genes of SDE miRNAs were enriched in categories and pathways related to fatty acid biosynthesis, transcriptional regulation, and MAPK as well as PI3K-Akt pathways. The expression of miR-206 was 36-fold higher in IM adipocytes as compared to those of SC adipocytes. The role of miR-206 is to regulate adipocyte proliferation through star-related lipid transfer domain containing 7 (*STARD7*) and it represses kruppel-like factor 4 (*KLF4*) expression which results in inhibition of adipogenesis. Thus, miR-206 acts as a suppressor of adipogenesis, and its attenuation can help in the production of pork with higher IMF [107]. Recently, Sun et al [108] analyzed the miRNA sequencing of high (2.94% ± 0.04%) and low (1.62%±0.02%) IMF group samples from Yorkshire pigs. From a total of 268 identified miRNAs, 28 were differentially expressed between two groups. From these 28 differentially expressed miRNAs, 13 were upregulated and 15 were downregulated. Top five differentially expressed miRNAs included miR-365-3p, miR-208b, miR-206, miR-126-5p, and miR-10a-5p, from which only miR-365-3p was highly expressed in low IMF group while others were high in high IMF group. Additionally, the authors reported that hsa-miR-208a-3p only expressed in the high IMF group, whereas has-miR-500a-5p was found only in the low IMF group [108].

Moreover, miR-34a targets the 3′UTR of Acyl-CoA synthetase long-chain family member 4 (*ACSL4*). Pig preadipocytes show the expression of *ACSL4* throughout the differentiation process. The transfection of miR-34a mimic tends to reduce the lipid droplet synthesis during adipogenesis while miR-34a inhibition increases the chances of lipid droplet formation. The expression miR34a target genes which included genes related to lipogeneses such as *ACSL4*, peroxisome proliferator-activated receptor γ (*PPARγ*), adipocyte protein 2 (*aP2*), and sterol regulatory element-binding protein-1C (*SREBP-1C*) was reduced upon overexpression of miR-34a while, downregulation of miR-34a had opposite results [109]. In addition to this mechanism for regulating the adipogenesis, miR-34a also inhibits the adipogenesis activity of platelet-derived growth factor receptor A (*PDGFRα*), a positive regulator of adipogenesis and IMF deposition in pigs, and controls the expression of lipogenesis genes *PPARg* and fatty acid binding protein 4 (*FABP4*) [110]. The miR-125-5p was found to regulate the proliferation and differentiation of porcine intramuscular preadipocytes. The overexpression of miR-125-5p in porcine preadipocytes enhanced the proliferation and reduced the differentiation of porcine IM preadipocytes while the downregulation of miR-125-5p showed opposite results. This effect was due to the targeting of porcine *KLF13* and *ELOVL* fatty acid elongase 6 (*ELOVL6*), a regulator of fatty acid composition, through miR-125-5p [101]. Further, miR-146a-5p acts as a potential regulator of porcine IMF adipogenesis. The mimics of miR-146a-5p tend to inhibit the proliferation and differentiation of porcine IM preadipocytes while miR146a-5p inhibitors augment the cell proliferation differentiation of preadipocytes. The proposed mechanism for regulation of preadipocyte proliferation by miR146a-5p is through targeting of *SMAD* family member 4 which diminishes TGF-β signaling. While the differentiation of adipocytes controlled through targeting tumor necrosis factor (*TNF*) receptor-associated factor 6 (*TRAF6*) attenuates the nuclear factor kappa beta (*NF-κB*) signaling. This miRNA could also be considered as a biomarker for amending the IM adipogenesis to improve pork quality [111]. The overexpression of miR-181a promoted the accumulation of lipid droplets, raised the amount of triglycerides, and decreased the *TNF-α* protein expression in porcine preadipocytes, while the inhibition of this miRNA showed opposite effects [112]. Another recent study reported that miR-181a promotes the differentiation of porcine preadipocytes by targeting *TGFβR1* [113]. In addition, the expression of miR-185 was found to gradually increase during 3T3-L1 cell differentiation. The overexpression of miR-185 led to the suppression of adipogenic markers, *PPARγ*, *FABP4*, fatty acid synthase (*FAS*), and lipoprotein lipase (*LPL*), and reduced the lipid accumulation in 3T3-L1 cells. while the inhibition of miR-185 promoted the differentiation of 3T3-L1 cells. This effect was due to the targeting of the *SREBP-1* gene by miR-185 [114]. Another miRNA, miR-425-5p also regulates the adipogenic differentiation of IM preadipocytes. The overexpression of miR-425-5p inhibits the IM adipogenic differentiation along with the downregulation of adipogenic marker genes *PPARγ*, *FABP4*, and fatty acid synthase. While the inhibitors of miR-425-5p augments adipogenesis. The inhibitory effect of miR425-5p on IM preadipocyte proliferation and adipogenesis is through repression of *cyclin B* and *KLF13* respectively [115]. These findings are in addition to the list of miRNAs for regulating the lipogenesis and ultimately the IMF deposition in skeletal muscles of pig. The summary of roles of aforementioned miRNAs in IMF in porcine skeletal muscles is given in Table 2.

## CONCLUSION AND PROSPECTS

Myogenesis in farm animals is directly linked to the production of meat and its overall quality. To date, the molecular mechanisms involved in governing the muscle development and deposition of fat in muscles to increase the quality of meat produced are not much understood. In recent years, miRNAs were found to play important roles in regulating the expression of some important genes and transcription factors involved in muscle development and IMF deposition in pigs. miRNAs could have significant influences on muscle traits, and play key roles in the phenotypic variation of the porcine muscle. However, underlying genetic factors and functional validation of each miRNA-target relationship remain to be determined. Thus, understanding the molecular mechanisms of myogenesis can turn out to be the breakthrough for increased meat production for meeting global food demands.

## Figures and Tables

**Table 1:** Different miRNAs and their roles in porcine skeletal muscle development

miRNA	Study model	Main findings	Targets	Reference
miR-1	Landrace and Tongcheng Pig (*in vivo*)	miR-1 targets *CNN3* gene related to skeletal muscle growth	*CNN3*	[73]
	Berkshire, Landrace, and Yorkshire pigs (*in vivo*)	Two miR-1 SNPs were responsible for regulating the type I and type II myofiber composition and number	*HDAC4* and *MEF2*	[74]
miR-21	Tongcheng pigs (*in vivo*)	Involves in skeletal muscle development by regulating the *PI3K/Akt/mTOR* signaling through targeting the *TGFβI* gene	*TGFβI*	[18]
miR-22	PSCs (*in vitro*)	miR-22 inhibits proliferation and promotes the differentiation of PSCs	*Cyclin D1, B1, p21, MyoG* and *MyHC*	[116]
miR-27a	Porcine myoblast cells (*in vitro*)	Control the myoblast differentiation by regulating Akt/FoxO1 signal pathway through targeting *MyoG*	*MyoG*	[75]
miR-27b	PSCs (*in vitro*)	miR-27b promotes the myogenesis in pigs by targeting *MDFI* in PSCs	*MDFI*	[76]
miR-34c	PSCs (*in vitro*)	miR-34c repress porcine muscle development by forming a regulatory loop with Notch1	*Notch1*	[77]
miR-127	PSCs (*in vitro*)	miR-127 decreases the PSCs proliferation and differentiation	*Cyclin B, D, PCNA, MyoG*, and *MyHC*	[61]
miR-143-3p	Hybrid male pigs (Landrace×Large White×Duroc)	miR-143-3p regulates skeletal myofiber differentiation by regulating the *MYH7* through *HDAC4-MEF2* pathway	*HDAC4, MyoD*, and *FLNC*	[78]
miR-155	Landrace and Tongcheng pigs (*in vivo*)	miR-155 regulates swine prenatal myogenesis by targeting *OLFML3*	*OLFML3*	[79]
miR-199b	PSCs (*in vitro*)	miR-199b promotes the proliferation of PSCs through activating Notch1 signal pathway by targeting *JAG1*	*JAG1*	[80]
miR-206	Tongcheng pigs (*in vivo*) and PIEC (*in vitro*)	Expression of the *SFRP1* gene is regulated by miR-206 which affects porcine skeletal muscle development	*SFRP1*	[69]
miR-208b	Berkshire, Landrace, and Yorkshire pigs (*in vivo*)	SNP of miR-208b affects myofiber characteristics and pork quality	*SOX-6* and *MYH7*	[63]
miR-378	PIEC (*in vitro*)	miR-378 regulates porcine skeletal muscle development by targeting *MAPK1* and *BMP2*	*MAPK1* and *BMP2*	[12]
miR-432	Landrace pigs (*in vivo*)	miR-432 involves in muscle development by targeting *MyoZ1*	*MyoZ1*	[81]

*CNN3*, calponin 3; *HDAC4*, histone deacetylase 4; *MEF2*, myocyte enhancer factor-2; *TGFβI*, transforming growth factor-β I; PSCs, porcine satellite cells; *Cyclin D1*, *B1*, *Cyclin D1*, *Cyclin B1*; *p21*, cyclin-dependent kinase inhibitor 1; *MyoG*, myogenin; *MyHC*, myosin heavy chain; *MDFI*, *MyoD* family inhibitor; *Notch1*, notch homolog 1; *PCNA*, proliferating cell nuclear antigen; *MyoD*, myoblast differentiation; *FLNC*, Filamin-C; *OLFML3*, olfactomedin-like protein 3; *JAG1*, jagged1; *SFRP1*, secreted frizzled-related protein 1; *PIEC*, porcine iliac endothelial cells; *SOX-6*, *SRY*-box transcription factor 6; *MYH7*, beta-myosin heavy chain; *MAPK1*, mitogen-activated protein kinase 1; *BMP2*, bone morphogenetic protein 2; *MyoZ1*, myozenin 1.

**Table 2:** Different miRNAs involved in intramuscular fat deposition in porcine skeletal muscles

miRNA	Study model	Main findings	Targets	Reference
miR-34a	Porcine primary IM preadipocytes (*in vitro*)	This miRNA acts as a regulator of IMF deposition in pigs	*ACSL4*, *PPARγ*, *aP2*, and *SREBP-1C*	[109]
	Porcine IM preadipocytes (*In vitro*)	miR-34a regulates IMF deposition through targeting *PDGFRα* gene	*PDGFRα*, *PPARg*, and *FABP4*	[110]
miR-125-5p	Porcine IM preadipocytes (*in vitro*)	This miRNA acts as a novel regulator for IMF deposition through regulating the proliferation and differentiation of porcine IM preadipocytes	*KLF13* and *ELOVL6*	[101]
miR-146a-5p	Porcine IM preadipocytes	miR-146a-5p regulates the IMF deposition through controlling the proliferation and differentiation of porcine IM preadipocytes	*SMAD4* and *TRAF6*	[111]
miR-206	Porcine IM and SC adipocytes (*in vitro*)	miR-206 suppresses the adipogenesis in porcine IM and SC adipocytes	*KLF4* and *STARD7*	[107]
miR-425-5p	Porcine IM preadipocytes (*in vitro*)	miR-425-5p regulates the proliferation and adipogenic differentiation of IM preadipocytes	*PPARγ*, *FABP4*, *FASN*, *Cyclin B*, *E*, and *KLF13*	[115]

IM, intramuscular; IMF, intermuscular fat; *ACSL4*, acyl-CoA synthetase long-chain family member 4; *PPARγ*, peroxisome proliferator-activated receptor gamma; *aP2*, adipocyte protein 2; *SREBP-1C*, sterol regulatory element-binding protein; *PDGFRα*, platelet-derived growth factor receptor; *PPARg*, peroxisome proliferator-activated receptor gamma; *FABP4*, fatty acid-binding protein; *KLF13*, kruppel like factor 13; *ELOVL6*, ELOVL fatty acid elongase 6; *SMAD4*, SMAD family member 4; *TRAF6*, TNF receptor-associated factor 6; SC, subcutaneous.
